# Mr. Merriman, on Dr. Dewee's Case of Midwifery

**Published:** 1807-11

**Authors:** Samuel Merriman

**Affiliations:** Curzon Street, May Fair


### *To the Editors of the Medical and Physical Journal.*

GENTLEMEN,

THE last Number of your Journal, page 337, records a case in Midwifery, under the care of Dr. Dewees and Dr. Jones, in which a most powerful and dangerous remedy was tried, apparently without the least necessity, and certainly with no good effect.

However unpleasant it may be to animadvert upon cases furnished by contemporary practitioners, it is necessary, for the improvement of our art, to examine critically whatever is published concerning it, and to express our disapprobation of any method, which seems to have been unnecessarily introduced, or injudiciously conducted. This motive alone, induces me to make a few remarks on the first of Dr. Dewee's cases.

In drawing up medical histories, there is some difficulty in discriminating between too much minuteness of detail, and too much generality; in the case under consideration, something more of circumstantial detail would have been an advantage.

Dr. Dewees has omitted mentioning two very essential particulars, namely, what was the age of the woman, and whether it was her first child; from a knowledge of these two facts, much assistance would have been afforded, in judging of the method of treatment.

Enough however is recorded, to render it manifest that the poor woman's labour would necessarily be tedious; for the waters had been evacuated (whether designedly is not said) early in her labour, and at the end of sixteen hours the os uteri was *very rigid*, and *but little opened*, and the os externum *scarcely large enough to admit a finger*. In such a state of the parts, no injury could have ensued from at least eight hours further delay; and surely no possible sible necessity could then exist for keeping a steady pressure against the perinæum, in order to prevent the escape of the child's head through it.

Dr. Dewees says, that the pains were frequent and brisk, but that there was not the least disposition in the soft parts to dilate; of course the pains, if at all uterine, were partial, and probably spasmodic. Such frequent and inefficacious pains (familiarly called cramp pains) almost constantly happen, when the anterior part of the uterus is squeezed between the child's head and the ossa pubis, as was evidently the situation here; for the child's head, covered by the uterus, was low down in the pelvis, and the waters had been long evacuated.

In such circumstances, the patient is extremely restless, and very urgent to get relief, and the by-standers are frequently very importunate to have assistance given; but more judgment is shown in soothing the patient and gaining time, than by any manual endeavours to hasten delivery.

I cannot conceive a case, in which this plan would be more required, than in the one we are now considering. The loss of some blood, according to the strength of the patient, an emollient clyster or two, perhaps even a dose of Castor oil, or some other mild laxative, and from thirty to forty drops of laudanum, with light and simple nourishment, joined to time and patience, would have overcome all the rigidity complained of; and the woman would have brought forth her child with very little assistance from her medical attendants.

It was however deemed expedient to adopt more active measures for overcoming the rigidity; and as tobacco clysters have been occasionally found useful by their relaxing qualities, in promoting a reduction of strangulated herniæ, it was determined to try whether they would not be equally efficacious in relaxing the rigidity of the os uteri. Accordingly, a *strong infusion of Tobacco* was thrown up the rectum, by which great sickness, vomiting, and fainting were excited; but though these distressing symptoms continued for an hour and a half, the desired relaxation did not take place. Another trial of the same remedy was then made, *much against the patient's will*; but though all the above enumerated symptoms were once more brought on, the rigidity of the os uteri continued as firm as ever.

I cannot however but think, that these clysters were, indirectly, productive of more effect than is attributed to them them by Dr. Dewees; for the very complete and sudden relaxation, which was produced immediately afterwards by drawing away ten ounces of blood, is a very unusual occurrence. It is to be recollected, that the os uteri was *very little open, and very rigid*; the child's head low in the pelvis, *covered by the uterus*; and the os externum *scarcely large enough to admit a finger*; yet on taking away ten ounces of blood, all the tightness and rigidity give way at once, the os and cervix uteri dilate and disappear, and opportunity is allowed for applying the forceps, by means of which a fine child is brought into the world *in a few minutes.*

Nothing but the most extreme debility could have produced so sudden a change in the state of the labour; it was like the delivery of a child, when the uterus has been relaxed by profuse hæmorrhage, or when the mother is rapidly sinking in the last stage of a consumption. Such a state of debility could not but be very dangerous and alarming; and I must think, was particularly to be guarded against, in a woman *subject to violent cholera morbus and convulsions,* by which diseases, we are told, she was carried off, about six days after her delivery.

Altogether, it is a melancholy case. From the detail given, there does not appear to have been more difficulty than might be remedied and overcome by the usual and authorized modes of assistance. In labours attended with difficulty, on account of the tightness and rigidity of the soft parts, sixteen hours afford but a short time for the uterine pains to produce a relaxation and dilatation of the os uteri and os externum, even if the membranes remain unbroken. But on this occasion, the waters had been evacuated early in the labour, and experience has taught us, that twenty, thirty, and forty hours must sometimes pass away, when such an accident has occurred, before the parts will be in a proper state to admit of the mother's safe delivery.

At all events, it has now been ascertained by two adequate trials, that tobacco clysters cannot be safely and advantageously employed, for relaxing the passages during a hard labour: it is therefore to be hoped, that no similar experiments will be made with such harsh and unmanageable medicines.

In another part of your Journal, p. 344, a case is related by Dr. Elmer, supposed to have been "a mortification and separation of the body of the uterus." Many histories are to be found in authors, nearly similar to Dr. Elmer's, mer's, which have proved to be in reality, cases of uterine polypus protruded through the os externum.

In the valuable Sepulchretum Anatomicum of Bonetus, several of this kind may be referred to. In some of these, the sphacelated tumour, supposed to be the uterus, was removed, and in others the putrefying mass occasioned the patient's death, but on opening the bodies, post mortem, the wombs were found entire, and in their proper situation within the pelvis. *Vide Boneti Sepulch. Anatom. tom. iii. lib. 3. sect. 32, obs. 7, 8, &c.*

It is scarcely credible, that a disease of so serious a nature as a mortification of the womb could have been cured by the very insufficient means, which, according to Dr. Elmer's account, were alone employed in the case he has communicated, viz. fomentations of bitter herbs, with large doses of a nitrous julep, and spir. lavend. comp.—Besides, if the whole uterus were extracted, what would become of the vesica urinaria and intestines. For want of their usual support, they must fall into the cavity of the pelvis, and would soon be protruded also, and death must inevitably be the consequence.

I am, &c.

SAMUEL MERRIMAN.

*Curzon Street, May Fair, Oct. 3. 1807.*

